# Furosemide use and survival in patients with esophageal or gastric cancer: a population-based cohort study

**DOI:** 10.1186/s12885-019-6242-8

**Published:** 2019-10-29

**Authors:** Peipei Liu, Úna C. McMenamin, Andrew D. Spence, Brian T. Johnston, Helen G. Coleman, Chris R. Cardwell

**Affiliations:** 1 Centre for Public Health, Queen’s University Belfast, Institute for Clinical Science, Royal Victoria Hospital, Grosvenor Road, Belfast, Belfast, Northern Ireland BT12 6BJ UK; 2 Belfast Health and Social Care Trust, Belfast, Northern Ireland UK; 3 Centre for Cancer Research and Cell Biology, Queen’s University Belfast, Belfast, Northern Ireland UK

**Keywords:** Epidemiology, Pharmacoepidemiology, Furosemide, Esophageal cancer, Gastric cancer, Mortality, Survival

## Abstract

**Background:**

Pre-clinical studies have shown that furosemide slows cancer cell growth by acting on the Na-K-2Cl transporter, particularly for gastric cancer cells. However, epidemiological studies have not investigated furosemide use and mortality in gastroesophageal cancer patients. Consequently, we conducted a population-based study to investigate whether furosemide use is associated with reduced cancer-specific mortality in esophageal/gastric cancer patients.

**Methods:**

A cohort of patients newly diagnosed with esophageal or gastric cancer between 1998 and 2013 were identified from English cancer registries and linked to the Clinical Practice Research Datalink to provide prescription records and the Office of National Statistics to provide death data up to September 2015. Time-dependant Cox-regression models were used to calculate hazard ratios (HRs) comparing cancer-specific mortality in furosemide users with non-users. Analyses were repeated restricting to patients with common furosemide indications (heart failure, myocardial infarction, edema or hypertension) to reduce potential confounding.

**Results:**

The cohort contained 2708 esophageal cancer patients and 2377 gastric cancer patients, amongst whom 1844 and 1467 cancer-specific deaths occurred, respectively. Furosemide use was not associated with reduced cancer-specific mortality overall (adjusted HR in esophageal cancer = 1.28, 95% CI 1.10, 1.50 and in gastric cancer = 1.27, 95% CI 1.08, 1.50) or when restricted to patients with furosemide indications before cancer diagnosis (adjusted HR in esophageal cancer = 1.07, 95% CI 0.88, 1.30 and in gastric cancer = 1.18, 95% CI 0.96, 1.46).

**Conclusions:**

In this large population-based cohort study, furosemide was not associated with reduced cancer-specific mortality in patients with esophageal or gastric cancer.

## Background

Esophageal and gastric cancer are the seventh and fifth most commonly diagnosed cancers worldwide, accounting for around 509,000 and 783,000 deaths annually [1]. The prognosis of esophageal and gastric cancer is poor even in developed countries, for instance in the United Kingdom (UK) the 5-year survival rate for both is under 20% [2], highlighting the importance of investigating new treatment options.

Furosemide, a loop diuretic, is commonly prescribed for patients with pulmonary edema caused by left ventricular heart failure and chronic heart failure [3, 4], and is also used to treat resistant edema and resistant hypertension through increasing urine production [5, 6]. Furosemide acts on the thick ascending limb of the loop of Henle, where it can block the luminal Na-K-2Cl (NKCC) transporter, a protein that transports sodium, potassium, and chloride between intracellular and extracellular fluid, thereby changing the osmotic pressure to increase the urine production [7, 8]. NKCC are found with two subtypes, ubiquitous NKCC1 and kidney-specific NKCC2, both of which are sensitive to furosemide [9].

Recently, evidence has emerged that the NKCC plays an important role in cancer cell growth. It has been shown that overexpression of the NKCC can induce cell proliferation [10, 11]. An in-vitro cell study found that NKCC1 expression was three times higher in poorly differentiated compared with moderately differentiated gastric adenocarcinoma cells [12]. This study also showed that furosemide reduced cell growth in poorly differentiated gastric adenocarcinoma cells, and suggested this action was via the inhibition of NKCC [12]. Consistently, NKCC1 was found to have higher expression in more poorly differentiated esophageal squamous-cell carcinoma (SCC) cases and depletion of NKCC1 inhibited cell proliferation [13]. Despite this preclinical evidence suggesting furosemide could slow gastroesophageal cancer progression, no studies have been conducted to investigate a potential association in humans.

Therefore, we conducted a large population-based cohort study to investigate whether furosemide use influences esophageal/gastric cancer-specific mortality.

## Methods

### Data source

Clinical Practice Research Datalink (CPRD) database is a primary care research database which covers nearly 11.3 million patients, accounting for approximately 6.9% of the UK population, and captures diagnoses, prescriptions and demographic information [14]. We obtained data from CPRD that was linked to the National Cancer Data Repository (NCDR) and the Office of National Statistics (ONS) mortality data. The NCDR contains cancer information from all English cancer registers, capturing diagnosis date, tumor characteristics and treatments. The ONS mortality data records cause and date of death.

Ethical approval for all purely observational research using CPRD data has been obtained from the East Midlands—Derby Research Ethics Service Committee (reference number: 05/MRE04/87). The study protocol was approved by The Independent Scientific Advisory of the Clinical Practice Research Datalink in 2015 (protocol number: 15_096RMn3).

### Study design and population

A cohort of newly diagnosed esophageal and gastric cancer patients was ascertained from English cancer registries between 1998 and 2013 using International Classification of Disease (ICD) codes C15 and C16, respectively. Individuals with previous cancer (apart from non-melanoma skin cancer or in situ tumors) were excluded using a list of Read codes modified for use in the CPRD database [15]. All eligible cases were further classed as adenocarcinoma (using ICD for Oncology (ICD-O) morphology codes 8140–8573) or SCC (ICD-O 8050–8082). Deaths from esophageal or gastric cancer were identified up to September 2015 based upon the underlying cause of death (from the ONS mortality data) using ICD codes C15, C16 and C26. Therefore, participants were followed until the earliest following event occurred: 1) participants ended their registration with the general practitioner (GP); 2) last data collection from GP; 3) patients died; 4) September 2015 at which time ONS follow-up ended.

### Definition of exposure

Furosemide was identified within GP prescription records based upon the British National Formulary. Overall, 78% of furosemide prescriptions were 40 mg, 20% were 20 mg. The daily defined doses (DDD) in each prescription were calculated by multiplying the quantity by the strength (in mg) and dividing by the mg in a DDD from the World Health Organization [16].

### Covariates

Lifestyle risk factors were obtained from GP records, including smoking status (never, former, current), alcohol consumption (never, former, current), and body mass index (BMI in kg/m^2^ categorised as: underweight < 18.5; normal weight 18.5 to 25; overweight: 25 to 30 and obese: ≥ 30). For these lifestyle risk factors, we extracted the closest records before esophageal/gastric cancer diagnosis, and ignored records more than 10 years before the diagnosis. Comorbidities at any time before cancer diagnosis were identified from the CPRD database according to the Charlson Comorbidity Index, including AIDS, cerebrovascular disease, chronic pulmonary disease, congestive heart disease, dementia, diabetes, diabetes with complications, hemiplegia, mild liver disease, moderate liver disease, myocardial infarction (MI), peptic ulcer disease, peripheral vascular disease, renal disease and rheumatological disease [15]. Deprivation was determined from the patients’ postcode based on the 2010 Index of Multiple Deprivation Score [17]. Also, furosemide indications including heart failure (HF) [18], MI [19], edema (Read code categories listed in Table 5 in Appendix) and hypertension [19] prior to cancer diagnosis were identified from the CPRD database using Read codes. Statin and aspirin use after diagnosis were also identified from GP records as previous studies have suggested these drugs could reduce mortality in patients with gastric or esophageal cancer [20, 21]. Patients using antihypertensive medications (diuretics, vasodilator antihypertensive drugs, centrally acting antihypertensive drugs, alpha-adrenoceptor blocking drugs, beta-blockers, angiotensin converting enzyme inhibitors, angiotensin receptor blockers, renin inhibitors and calcium channel blockers) were identified from prescription records [22].

### Statistical analysis

Summary statistics and frequencies were determined for patient characteristics by furosemide use.

**Fig. 1:**
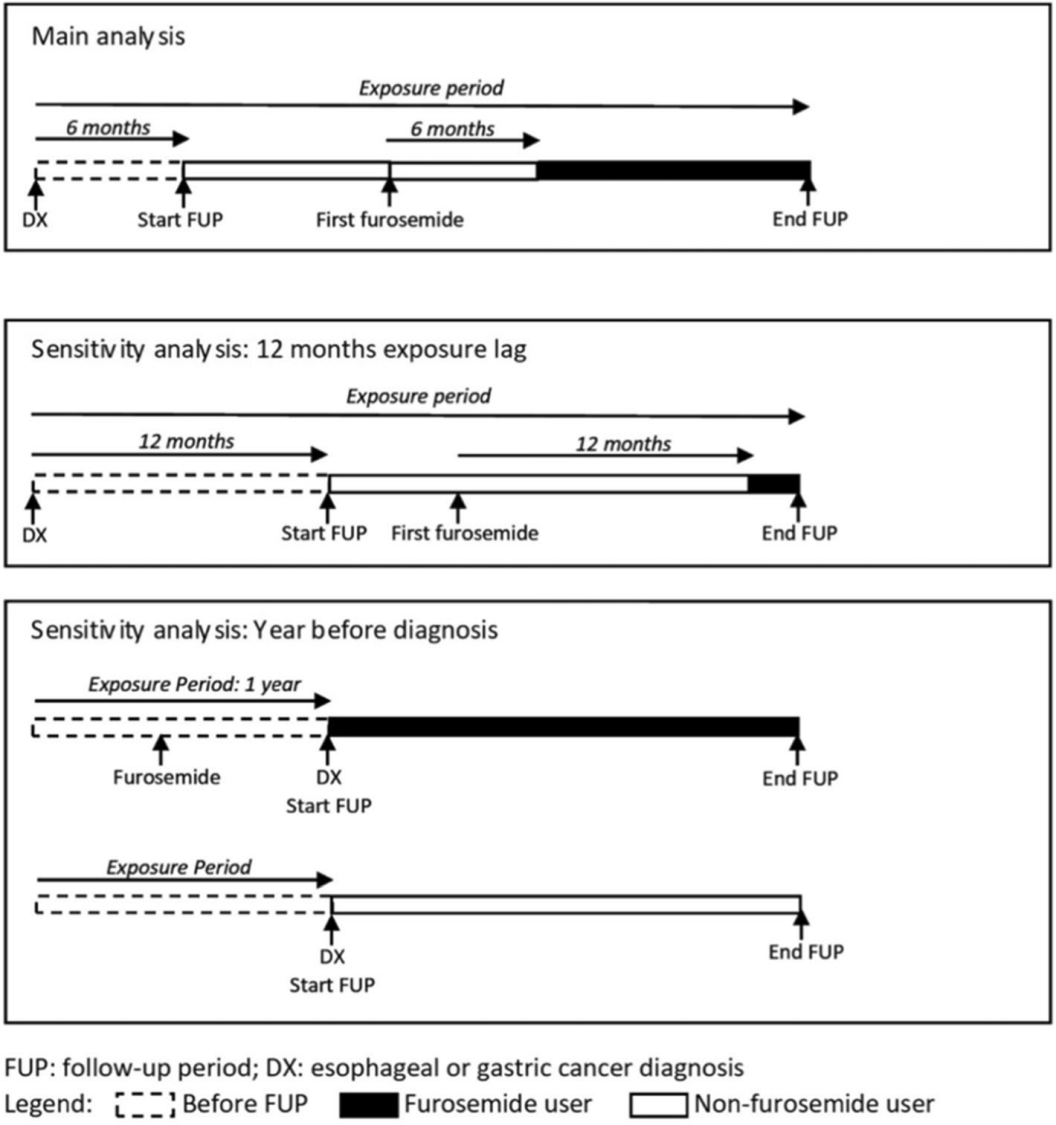
Figure illustrating the study design in the main and sensitvity analyses of furosemide and cancer-specific mortality

In the primary analyses, patients who died within 6 months were excluded as it seems unlikely that medication use after diagnosis could benefit such patients. Therefore, individuals were followed from 6 months after diagnosis to cancer-specific death or censoring. Furosemide use was treated as a time-varying covariate, to avoid immortal time bias [23]. We also utilised a lag, as recommended, to remove prescriptions in the period immediately prior to death in order to minimise potential reverse causation [24]. The study design is illustrated in Fig. 1. Individuals were regarded as furosemide non-users until 6 months after their first prescription, at which point they were considered furosemide users until the end of follow-up. Hazard ratios (HR) and 95% confidence intervals (CI) were calculated using Cox regression models before and after adjustment for relevant confounders. The main model contained the following confounders: year of diagnosis, age at diagnosis, sex, deprivation, comorbidities (cerebrovascular disease, chronic pulmonary disease, congestive heart disease, diabetes, myocardial infarction, peptic ulcer disease, peripheral vascular disease, renal disease, rheumatological disease and liver disease), post-diagnosis statin or aspirin use as time-varying covariates as furosemide, and cancer treatment (surgery, radiotherapy and chemotherapy within 6 months after diagnosis). An exposure-response analysis was calculated using a time-varying covariate to account for the dose and duration of use. In which, we categorised the patients as non-users (as described above), short-term users between 6 months after their first prescription and 6 months after their 365th DDD (or 12th prescription, approximately corresponding to 1 year of issued medication) and long-term users after that time. These analyses were repeated restricting the cohort to patients with any pre-diagnosis furosemide indications, described above, to attempt to reduce the impact of confounding by indication [25]. All of these analyses were conducted separately in esophageal and gastric cancer patients.

### Sensitivity and subgroup analysis

Various sensitivity and subgroup analyses were conducted. We performed an analysis changing the outcome to cause of death from any cancer, and any cause. Subgroup analyses were conducted by the main histological subtypes (adenocarcinoma and SCC), among patients who underwent surgery within 6 months of diagnosis and by year of cancer diagnosis. We conducted an analysis additionally adjusting for variables which were incompletely recorded such as lifestyle exposure (smoking and alcohol conjunctively, and BMI independently), and tumor characteristics (stage and grade, separately). We conducted an analysis restricted to patients with any hypertensive medications use in the year prior to cancer diagnosis, where we removed those cases whose record period before diagnosis were less than 1 year. To further investigate potential confounding by hypertension (the most common furosemide indication before diagnosis) we conducted an active comparator analysis in which we compared furosemide users after diagnosis to users of other antihypertensive medications after diagnosis, based upon a time-varying covariate with a 6 month lag [22]. Also, we conducted an analysis extending the lag to 12 months, in which individuals who died within 12 months after diagnosis were excluded and follow-up started at 12 months. As pre-clinical evidence suggested furosemide would have a greater impact on more poorly differentiated tumor [12], we repeated the analysis restricted to patients diagnosed with high grade (3 or 4) tumors. We also repeated the analysis for all loop diuretics (furosemide, bumetanide and torasemide) as all of them have similar NKCC inhibitor properties.

Finally, to attempt to investigate the impact of furosemide on the developing tumor we conducted an analysis of furosemide use in the year before cancer diagnosis on cancer-specific mortality, in which we started to follow participants from the date of cancer diagnosis and did not exclude deaths in the first 6 months [23]. Patients with less than 1 year of records before cancer diagnosis were excluded.

## Results

Overall 4799 and 4537 newly diagnosed primary esophageal and gastric cancer patients were identified in CPRD. After exclusion of patients who died within 6 months of diagnosis, there were a total of 2708 esophageal cancer cases and 2377 gastric cancer cases included in the main analyses. In these patients, the median follow-up was 1.3 years (range 0.5 to 17.2 years) for esophageal cancer and 1.5 years (range 0.5 to 17.2 years) for gastric cancer. Patient characteristics are shown in Table 1. Furosemide users were more likely to be older (mean age at diagnosis was 75 for esophageal cancer and 77 for gastric cancer), have comorbidities (particularly HF, edema, MI, and hypertension), use statins or aspirin after diagnosis, have previously smoked and be obese (BMI ≥ 30 kg/m^2^) compared to non-users. Other characteristics were generally similar in furosemide users and non-users, see Table 1.

**Table 1:** Patients clinical characteristics by furosemide users and non-users

		Esophageal n (%)		Gastric n (%)	
		Users	Never-users	Users	Never-users
Year of diagnosis	1998–2002	85 (25%)	482 (20%)	116 (31%)	507 (25%)
	2003–2007	123 (36%)	789 (33%)	126 (34%)	719 (36%)
	2008–2013	135 (39%)	1094 (46%)	130 (35%)	779 (39%)
Age at diagnosis: mean		74.5	68.2	76.5	69.6
	< 60	25 (7%)	564 (24%)	21 (6%)	408 (20%)
	60–69	86 (25%)	755 (32%)	64 (17%)	506 (25%)
	70–79	124 (36%)	655 (28%)	150 (40%)	698 (35%)
	80+	108 (31%)	391 (17%)	137 (37%)	393 (20%)
Gender	Male	217 (63%)	1611 (68%)	241 (65%)	1368 (68%)
Deprivation quintile	1 (least deprived)	63 (18%)	501 (21%)	68 (18%)	381 (19%)
	2	86 (25%)	602 (25%)	93 (25%)	480 (24%)
	3	70 (20%)	469 (20%)	61 (16%)	413 (21%)
	4	71 (21%)	446 (19%)	81 (22%)	429 (22%)
	5 (most deprived)	53 (15%)	346 (15%)	69 (19%)	299 (15%)
Treatment	Surgery	125 (36%)	959 (41%)	181 (49%)	1029 (51%)
	Chemotherapy	116 (34%)	1178 (50%)	76 (20%)	784 (39%)
	Radiotherapy	108 (31%)	591 (25%)	30 (8%)	130 (6%)
Tumor type	Adenocarcinoma	214 (62%)	1391 (59%)	303 (81%)	1581 (79%)
	Squamous	99 (29%)	716 (30%)	<5^a^	19 (1%)
	Others	30 (9%)	258 (11%)	64-69^a^	405 (20%)
Grade	1	17 (7%)	92 (5%)	18 (7%)	69 (5%)
	2	131 (53%)	775 (44%)	103 (40%)	484 (33%)
	3	93-98^a^	892 (50%)	132-137^a^	892 (61%)
	4	<5^a^	16 (1%)	<5^a^	17 (1%)
	Missing	97	590	114	543
Stage	1	8 (17%)	36 (8%)	7 (16%)	33 (12%)
	2	10 (21%)	86 (18%)	9 (21%)	54 (20%)
	3	20 (43%)	208 (44%)	8 (19%)	67 (28%)
	4	9 (19%)	143 (30%)	19 (44%)	116 (43%)
	Missing	296	1892	329	1735
Other medication use^b^	Statin	133 (39%)	553 (23%)	164 (44%)	479 (24%)
	Aspirin	146 (43%)	423 (18%)	132 (35%)	366 (18%)
Smoking	Never	101 (43%)	701 (39%)	103 (39%)	609 (42%)
	Former	94 (40%)	621 (35%)	123 (46%)	510 (35%)
	Current	42 (18%)	473 (26%)	39 (15%)	318 (22%)
	Missing	106	570	107	568
Alcohol consumption	Never	36 (16%)	235 (15%)	51 (20%)	243 (18%)
	Former	7 (3%)	63 (4%)	12 (5%)	45 (3%)
	Current	184 (81%)	1322 (82%)	191 (75%)	1030 (78%)
	Missing	116	745	118	687
BMI	Underweight (< 18.5)	6 (3%)	68 (5%)	<5^a^	50 (4%)
	Normal (18.5–24.9)	63 (29%)	549 (38%)	64-69^a^	429 (35%)
	Overweight (25–29.9)	86 (39%)	531 (37%)	91 (39%)	504 (41%)
	Obese (≥30)	63 (29%)	300 (21%)	74 (32%)	238 (19%)
	Missing	125	917	138	784
Selected comorbidities	Heart failure	51 (15%)	58 (2%)	64 (17%)	61 (3%)
	Myocardial infarction	38 (11%)	124 (5%)	48 (13%)	115 (6%)
	Hypertension	187 (55%)	979 (41%)	217 (58%)	826 (41%)
	Edema	79 (23%)	185 (8%)	86 (23%)	148 (7%)

^a^Ranges presented for statistical disclosure control

^b^After cancer diagnosis, using a 6 months lag (same as furosemide)

As shown in Table 2, there was a slight increase in both esophageal and gastric cancer-specific mortality in users of furosemide compared with non-users in the primary analyses (adjusted HR in esophageal cancer = 1.28, 95% CI 1.10, 1.50 and in gastric cancer = 1.27, 95% CI 1.08, 1.50). After restricting to patients with identified indications for furosemide, to compare the furosemide users to more similar non-users, there was no significant association between furosemide use and cancer-specific mortality in patients with esophageal or gastric cancer (adjusted HR in esophageal cancer = 1.07, 95% CI 0.88, 1.30 and in gastric cancer = 1.18, 95% CI 0.96, 1.46). Similarly, in analyses accounting for dose and duration of use in the restricted cohort, the adjusted HRs for over 1 year of furosemide use (i.e. 365 DDDS) were 1.27 (95% CI 0.86, 1.88) and 1.34 (95% CI 0.93, 1.96) in esophageal and gastric cancer patients, respectively (Table 2).

**Table 2:** Association between furosemide use after diagnosis and esophageal or gastric cancer mortality

	All patients						Restricted to any diagnosis of hypertension/edema/MI/HF^a^					
	Patients	Deaths	Person years	Unadjusted HR (95% CI)	Adjusted^b^ HR (95%CI)	*P*-value (trend)	Patients	Deaths	Person years	Unadjusted HR (95% CI)	Adjusted^b^ HR (95%CI)	*P*-value (trend)
Esophageal												
Non-user	2365	1625	4336	1.00 (ref. cat.)	1.00 (ref. cat.)		1097	763	1747	1.00 (ref. cat.)	1.00 (ref. cat.)	
User	343	219	471	1.47 (1.27, 1.69)	1.28 (1.10, 1.50)	0.002	233	148	285	1.29 (1.08, 1.55)	1.07 (0.88, 1.30)	0.502
1–12 prescriptions	270	184	361	1.40 (1.20, 1.63)	1.23 (1.04, 1.45)	(< 0.001)	182	122	227	1.23 (1.02, 1.50)	1.03 (0.83, 1.26)	(0.267)
≥ 12 prescriptions	73	35	110	2.02 (1.44, 2.84)	1.74 (1.22, 2.49)		51	26	59	1.69 (1.13, 2.52)	1.41 (0.92, 2.16)	
1–365 DDDs	266	180	347	1.42 (1.21, 1.65)	1.25 (1.06, 1.47)	(0.001)	175	117	214	1.24 (1.02, 1.51)	1.03 (0.84, 1.28)	(0.326)
≥ 365 DDDs	77	39	124	1.76 (1.28, 2.43)	1.52 (1.08, 2.13)		58	31	71	1.53 (1.06, 2.21)	1.27 (0.86, 1.88)	
Gastric												
Non-user	2005	1268	4558	1.00 (ref. cat.)	1.00 (ref. cat.)		906	541	1978	1.00 (ref. cat.)	1.00 (ref. cat.)	
User	372	199	612	1.38 (1.19, 1.60)	1.27 (1.08, 1.50)	0.004	264	138	440	1.36 (1.13, 1.64)	1.18 (0.96, 1.46)	0.125
1–12 prescriptions	263	161	382	1.38 (1.17, 1.63)	1.26 (1.06, 1.50)	(0.005)	181	108	274	1.31 (1.07, 1.62)	1.14 (0.91, 1.43)	(0.079)
≥ 12 prescriptions	109	38	230	1.37 (0.99, 1.91)	1.35 (0.96, 1.91)		83	30	166	1.56 (1.07, 2.29)	1.38 (0.92, 2.07)	
1–365 DDDs	259	155	364	1.39 (1.18, 1.65)	1.26 (1.06, 1.51)	(0.006)	175	102	251	1.32 (1.07, 1.63)	1.14 (0.90, 1.43)	(0.080)
≥ 365 DDDs	113	44	248	1.34 (0.99, 1.82)	1.32 (0.96, 1.83)		89	36	189	1.49 (1.05, 2.10)	1.34 (0.93, 1.96)	

^a^Restricted to patients with any diagnosis of hypertension, edema, myocardial infarction or heart failure at any time prior to esophageal or gastric cancer diagnosis.

^b^Adjusted for age at diagnosis, sex, year of diagnosis, deprivation, radiotherapy within 6 months, chemotherapy within 6 months, surgery within 6 months, comorbidities (prior to cancer diagnosis, including cerebrovascular disease, chronic pulmonary disease, congestive heart disease, diabetes, myocardial infarction, peptic ulcer disease, peripheral vascular disease, renal disease, rheumatological disease, liver disease), and other medication use (statins, aspirin, time-varying after diagnosis)

Sensitivity analyses generally showed similar associations to the main results (Tables 3 and 4). In particular, associations were similar when the use of furosemide in the year before diagnosis was investigated, when a 12 month lag was used, when stratified by histological tumor subtype, when stratified by year of diagnosis, when exposure was expanded to all loop diuretics, after additional adjustment for BMI, after additional adjustment for smoking and alcohol, after additional adjustment for tumor stage or grade, and when restricted to patients diagnosed with high grade tumors. The analyses were also similar when restricting to patients who were users of any antihypertensive medications in the year prior to cancer diagnosis, and when using an active comparator to compare users of furosemide to users of other antihypertensive medications after cancer diagnosis. There was a slight increase in all-cancer and all-cause mortality in users of furosemide, compared with non-users, for both esophageal and gastric cancer patients. Furthermore, there was a slight increase in cancer-specific mortality with furosemide use when restricted to surgically treated patients (Tables 3 and 4).

**Table 3:** Sensitivity and subgroup analyses for furosemide use and esophageal cancer-specific mortality

	Non-users			Users			Unadjusted HR (95% CI)	Adjusted^a^ HR (95% CI)
	Patients	Deaths	Person-years	Patients	Deaths	Person-years		
All patients^b^								
Main analysis	2365	1625	4336	343	219	471	1.47 (1.27, 1.69)	1.28 (1.10, 1.50)
All cancer death	2365	1730	4336	343	248	471	1.56 (1.36, 1.78)	1.34 (1.16, 1.55)
All cause death	2365	1784	4336	343	277	471	1.68 (1.47, 1.90)	1.44 (1.25, 1.65)
Use in the year before diagnosis	3862	2836	5739	567	438	469	1.46 (1.32, 1.62)	1.14 (1.02, 1.28)
12 month lag	1481	883	3401	208	112	336	1.56 (1.28, 1.90)	1.41 (1.13, 1.75)
Tumor type^c^								
Adenocarcinoma	1478	1015	2837	218	145	322	1.55 (1.30, 1.85)	1.30 (1.07, 1.57)
Squamous cell carcinoma	717	496	1214	99	59	115	1.38 (1.05, 1.81)	1.64 (1.18, 2.26)
Additionally adjusted for smoking and alcohol^d^	1391	1031	1581	182	134	173	1.34 (1.11, 1.60)	1.12 (0.91, 1.36)
Additionally adjusted for BMI^e^	1448	1036	1928	218	148	243	1.25 (1.05, 1.49)	1.09 (0.90, 1.32)
All loop diuretics^f^	2348	1613	4313	360	231	492	1.44 (1.25, 1.66)	1.24 (1.06, 1.44)
Additionally adjusted for stage^g^	473	285	641	47	26	62	1.24 (0.83, 1.86)	1.19 (0.74, 1.91)
Additionally adjusted for grade^h^	1775	1229	3131	246	164	312	1.55 (1.31, 1.82)	1.44 (1.20, 1.73)
Restricted to patients with high grade diagnosis^i^	908	661	1371	98	70	113	1.55 (1.21, 1.99)	1.36 (1.04, 1.79)
Restricted to patients surgically treated^j^	959	579	2460	125	70	248	1.64 (1.28, 2.11)	1.44 (1.10, 1.90)
Restricted to any hypertensive medication use^k^	1062	736	1676	264	174	335	1.34 (1.13, 1.58)	1.13 (0.94, 1.36)
Furosemide vs other antihypertensive medication^l^	1106	771	1909	343	219	471	1.46 (1.25, 1.70)	1.27 (1.07, 1.49)
Year of diagnosis^m^: 1998 to 2002	482	372	1095	85	58	131	1.72 (1.30, 2.27)	1.46 (1.06, 1.99)
2003 to 2007	789	601	1701	123	80	179	1.45 (1.15, 1.84)	1.28 (0.99, 1.65)
2008 to 2013	1094	652	1540	135	81	162	1.32 (1.05, 1.67)	1.22 (0.94, 1.59)
Restricted to any diagnosis of hypertension/edema/MI/HF^n^								
Main analysis	1097	763	1747	233	148	285	1.29 (1.08, 1.55)	1.07 (0.88, 1.30)
12 month lag	683	422	1314	138	75	194	1.31 (1.02, 1.68)	1.04 (0.79, 1.37)
Tumor type^o^								
Adenocarcinoma	715	493	1192	146	98	188	1.43 (1.14, 1.78)	1.08 (0.85, 1.39)
Squamous cell carcinoma	310	214	471	71	41	78	1.18 (0.85, 1.66)	1.32 (0.87, 2.00)
Additionally adjusted for smoking and alcohol^d^	758	567	785	137	97	137	1.15 (0.92, 1.43)	0.95 (0.74, 1.21)
Additionally adjusted for BMI^e^	774	562	900	162	107	182	1.03 (0.83, 1.27)	0.83 (0.65, 1.05)
All loop diuretics^f^	1082	752	1730	248	159	302	1.28 (1.08, 1.52)	1.03 (0.84, 1.25)
Additionally adjusted for Stage^g^	275	160	351	33	17	32	1.29 (0.78, 2.14)	1.37 (0.76, 2.48)
Additionally adjusted for Grade^h^	832	578	1328	172	112	211	1.41 (1.15, 1.72)	1.25 (0.99, 1.57)
Year of diagnosis^p^: 1998 to 2002	149	120	290	43	30	47	1.47 (0.98, 2.20)	1.07 (0.65, 1.77)
2003 to 2007	348	279	685	83	58	112	1.34 (1.01, 1.79)	1.12 (0.81, 1.54)
2008 to 2013	600	364	772	107	60	126	1.16 (0.88, 1.52)	0.99 (0.72, 1.36)

^a^Adjusted for age at diagnosis, sex, year of diagnosis, deprivation, radiotherapy within 6 months, chemotherapy within 6 months, surgery within 6 months, comorbidities (prior to diagnosis, including cerebrovascular disease, chronic pulmonary disease, congestive heart disease, diabetes, myocardial infarction, peptic ulcer disease, peripheral vascular disease, renal disease, rheumatological disease and liver disease), and other medication use (statins, aspirin, time-varying after diagnosis)

^b^Sensitivity analyses based on the primary main analyses, including all eligible patients except were indicated

^c^*P-*value for interaction for esophageal cancer is 0.794

^d^Restricted to patient with smoking and alcohol records

^e^Restricted to patient with BMI records

^f^Association between all loop diuretics (including furosemide, bumetanide and torasemide) use after diagnosis and gastric or esophageal cancer mortality

^g^Additionally adjusted for tumor stage

^h^Additionally adjusted for tumor grade

^i^Restricted to patients who were diagnosed as grade 3 or 4 cancer

^j^Restricted to patients who received the surgery treatment within 6 months of diagnosis

^k^Restricted to patients with any antihypertensive medication use in the year prior to cancer diagnosis

^l^Using other antihypertensive medications after cancer diagnosis as an active comparator

^m^*P*-value for interaction across cancer diagnosis year is 0.265

^n^Restricted to patients with any diagnosis of hypertension, edema, myocardial infarction or heart failure at any time prior to esophageal cancer diagnosis

^o^*P*-value for interaction for esophageal cancer is 0.524

^p^*P*-value for interaction across cancer diagnosis year is 0.964

**Table 4:** Sensitivity and subgroup analyses for furosemide use and gastric cancer-specific mortality

	Non-users			Users				
	Patients	Deaths	Person-years	Patients	Deaths	Person-years	Unadjusted HR (95% CI)	Adjusted^a^ HR (95% CI)
All patients^b^								
Main analysis	2005	1268	4558	372	199	612	1.38 (1.19, 1.60)	1.27 (1.08, 1.50)
All cancer death	2005	1405	4558	372	244	612	1.50 (1.31, 1.72)	1.37 (1.18, 1.59)
All cause death	2005	1512	4558	372	293	612	1.63 (1.44, 1.85)	1.44 (1.25, 1.65)
Use in the year before diagnosis	3584	2534	5693	608	431	573	1.32 (1.19, 1.46)	1.12 (1.00, 1.26)
12 month lag	1360	712	3733	234	101	464	1.35 (1.10, 1.67)	1.21 (0.96, 1.53)
Adenocarcinoma	1813	1182	4015	336	186	561	1.33 (1.14, 1.55)	1.25 (1.05, 1.49)
Additional adjusted for smoking and alcohol^c^	1091	780	1469	206	133	204	1.30 (1.08, 1.56)	1.41 (1.13, 1.75)
Additional adjusted for BMI^d^	1221	825	1984	234	135	282	1.27 (1.06, 1.53)	1.26 (1.02, 1.54)
All loop diuretics^e^	1984	1256	4514	393	211	656	1.35 (1.17, 1.56)	1.27 (1.08, 1.50)
Additionally adjusted for Stage^f^	270	155	409	43	18	66	0.88 (0.54, 1.44)	0.64 (0.35, 1.15)
Additionally adjusted for Grade^g^	1462	945	3316	258	134	422	1.31 (1.09, 1.57)	1.26 (1.03, 1.54)
Restricted to patients with high grade diagnosis^h^	909	610	1770	137	81	207	1.34 (1.06, 1.69)	1.37 (1.05, 1.79)
Restricted to patients surgically treated^i^	1029	556	3198	181	85	385	1.64 (1.30, 2.06)	1.74 (1.35, 2.23)
Restricted to any hypertensive medication use^j^	899	563	1914	283	149	434	1.31 (1.09, 1.57)	1.14 (0.93, 1.39)
Furosemide vs other antihypertensive medication^k^	953	593	2169	372	199	612	1.49 (1.27, 1.76)	1.31 (1.10, 1.57)
Year of diagnosis^l^: 1998 to 2002	507	365	1496	116	67	232	1.52 (1.17, 1.98)	1.50 (1.11, 2.02)
2003 to 2007	719	476	1779	126	76	211	1.60 (1.25, 2.04)	1.64 (1.24, 2.15)
2008 to 2013	779	427	1283	130	56	169	1.07 (0.81, 1.41)	1.01 (0.74, 1.38)
Restricted to any diagnosis of hypertension/edema/MI/HF^m^								
Main analysis	906	541	1978	264	138	440	1.36 (1.13, 1.64)	1.18 (0.96, 1.46)
12 month lag	615	288	1606	168	75	334	1.46 (1.13, 1.88)	1.22 (0.91, 1.64)
Adenocarcinoma	813	502	1721	235	130	393	1.33 (1.10, 1.62)	1.16 (0.93, 1.44)
Additionally adjusted for smoking and alcohol^c^	550	368	739	160	101	155	1.36 (1.09, 1.70)	1.36 (1.04, 1.77)
Additionally adjusted for BMI^d^	630	401	1013	177	99	223	1.24 (0.99, 1.55)	1.14 (0.89, 1.47)
All loop diuretics^e^	890	533	1949	280	146	470	1.34 (1.11, 1.61)	1.18 (0.96, 1.45)
Additionally adjusted for Stage^f^	129	68	154	33	51	46	1.01 (0.58, 1.78)	0.53 (0.24, 1.15)
Additionally adjusted for Grade^g^	659	408	1435	174	89	274	1.31 (1.04, 1.65)	1.11 (0.85, 1.45)
Year of diagnosis^n^: 1998 to 2002	148	108	447	69	35	160	1.24 (0.85, 1.83)	0.95 (0.59, 1.54)
2003 to 2007	325	208	809	86	56	141	1.65 (1.22, 2.22)	1.57 (1.11, 2.23)
2008 to 2013	433	225	722	109	47	140	1.17 (0.85, 1.60)	1.04 (0.72, 1.49)

^a^Adjusted for age at diagnosis, sex, year of diagnosis, deprivation, radiotherapy within 6 months, chemotherapy within 6 months, surgery within 6 months, comorbidities (prior to diagnosis, including cerebrovascular disease, chronic pulmonary disease, congestive heart disease, diabetes, myocardial infarction, peptic ulcer disease, peripheral vascular disease, renal disease, rheumatological disease and liver disease), and other medication use (statins, aspirin, time-varying after diagnosis)

^b^Sensitivity analyses based on the primary main analyses, including all eligible patients except were indicated

^c^Restricted to patient with smoking and alcohol records

^d^Restricted to patient with BMI records

^e^Association between all loop diuretics (including furosemide, bumetanide and torasemide) use after diagnosis and gastric cancer mortality

^f^Additionally adjusted for tumor stage

^g^Additionally adjusted for tumor grade

^h^Restricted to patients who were diagnosed as grade 3 or 4 cancer

^i^Restricted to patients who received the surgery treatment within 6 months of diagnosis

^j^Restricted to patients with any antihypertensive medication use in the year prior to cancer diagnosis

^k^Using other antihypertensive medications after cancer diagnosis as an active comparator

^l^*P-*value for interaction across cancer diagnosis year is 0.390

^m^Restricted to patients with any diagnosis of hypertension, edema, myocardial infarction or heart failure at any time prior to esophageal or gastric cancer diagnosis

^n^*P-*value for interaction across cancer diagnosis year is 0.070

## Discussion

In this large population-based cohort we did not find evidence that furosemide was associated with a reduced risk of cancer-specific or all-cause mortality in patients with esophageal or gastric cancer. In the primary analyses, we found furosemide use was associated with slightly increased mortality in patients with esophageal or gastric cancer. However, when we restricted the analyses to patients with furosemide indications, there was little evidence of any strong association between furosemide use and deaths from esophageal or gastric cancer. Findings were similar across most subgroup and sensitivity analyses.

This is, to date, the first observational study assessing the influence of furosemide use on esophageal/gastric cancer mortality. A limited number of studies have investigated furosemide use and cancer risk or survival, concentrated on investigations into breast, skin and lip cancer but not esophageal/gastric cancer, and furosemide was not the primary exposure of interest [26–28]. No consistent associations were observed for furosemide use and risk or survival of these cancers from human studies. Preclinical studies have recently shown that inhibition of the NKCC1 could slow cancer cell deterioration by influencing cancer cell growth and metastasis [11], and furosemide could reduce cell growth in poorly differentiated gastric adenocarcinoma cells [12]. In contrast, our study did not detect any evidence that users of furosemide either before or after diagnosis of esophageal or gastric cancer had reduced cancer-specific mortality. This lack of protective effect could reflect differences in drug dose, duration of use or the recognised difficulty of in vitro models to recreate the complexity of human carcinogenesis, physiology and progression [29].

The main strength of this study is that it utilised high quality data sources including cancer-registry records, ONS mortality data, and the CPRD for prescription data. In the UK, furosemide is only available through GP prescriptions and therefore these records are likely to capture all of the use in these populations [30]. The use of an electronic record of GP prescriptions also eliminated recall bias. In this study, we also had long-term follow-up, which makes it possible to assess the impact of furosemide on gastric or esophageal cancer prognosis long after diagnosis.

Confounding by indication is often encountered in pharmacoepidemiology studies as allocation of prescription is not randomized and the drug indication may be related to the outcomes of interest [25, 31]. We conducted a number of analyses to account for confounding by indication. Firstly, we conducted analyses restricted to patients with furosemide indications, restricted to patients with any antihypertensive medications use and an active comparator analysis comparing furosemide users to users of other antihypertensive drugs. In all of these analyses, no association between furosemide use and cancer-specific mortality was found. Although diuretics are sometimes used for malignant ascites, this is not likely to impact our results because furosemide is not commonly used for this purpose [32, 33]. Furthermore, application of the lag period removed prescriptions in the 6 months before death in the main analysis (and 12 months in sensitivity analysis), which would almost entirely remove use for ascites as survival with malignant ascites is very short [34]. Moreover, this potential weakness would not have impacted the analysis of furosemide use in the year before diagnosis, which also did not show evidence of improved survival with furosemide use.

Another potential weakness is that although we were able to adjust for a wide range of confounders there is the possibility of residual confounding by incompletely recorded variables such as cancer stage and lifestyle exposures. However, based on the available data, additional adjustment of some lifestyle exposures for which we had incomplete records did not change the main findings. Also, there was no difference when analysis was restricted to poorly differentiated cancers.

## Conclusion

In conclusion, in the first study to investigate furosemide use and survival in esophageal and gastric cancer patients, we did not find evidence that furosemide use was associated with improved survival. Further preclinical or observational studies should be designed to investigate this association according to stratified analyses by tumor stage or molecular characteristics to fully exclude a potential role for this medication as an adjuvant therapy.

## Data Availability

The data that support the findings of this study are available from CPRD database but restrictions apply to the availability of these data, which were used under license for the current study, and so are not publicly available. STATA code used to conduct analysis is available from the corresponding author on reasonable request.

## Appendix

**Table 5:** Edema code list

Medcode	Readcode	Readterm
11,396	R023400	[D] Peripheral oedema
3158	183..00	Oedema
6047	183..11	Oedema - symptom
1906	22C2.11	O/E - ankle oedema
4950	R023.00	[D] Oedema
6585	22C4.11	O/E - leg oedema
15,477	R023z11	[D] Dependent oedema
14,702	R023z00	[D] Oedema NOS
20,553	22C2.00	O/E - oedema of ankles
10,931	22C..00	O/E - oedema
19,358	22C4.00	O/E - oedema of legs
2140	183..12	Swelling - oedema - symptom
7321	H541z00	Pulmonary oedema NOS
1284	22C3.00	O/E - oedema of feet
30,309	183Z.00	Oedema NOS
9108	1837	Pitting oedema
558	H584.00	Acute pulmonary oedema unspecified
5155	23E1.00	O/E - pulmonary oedema
7106	22C3.11	O/E - foot oedema
6651	22C..11	O/E - swelling - oedema
28,419	22CZ.00	O/E - oedema NOS
5293	H584z00	Acute pulmonary oedema NOS
22,500	8E95.00	Reduction of oedema
20,301	R023000	[D] Oedema, generalized
43,618	G581.12	Pulmonary oedema - acute
102,627	183B.00	Worsening pulmonary oedema
22,734	1838	Sacral oedema
19,714	22C5.11	O/E - thigh oedema
9392	22C7.00	O/E - sacral oedema
31,747	22C6.00	O/E - abdominal oedema
61,224	22C5.00	O/E - oedema of thighs
26,082	H541000	Chronic pulmonary oedema
108,888	C366200	Idiopathic oedema
48,466	H584.11	Acute oedema of lung, unspecified

## Abbreviations

BMI: Body mass index
CIs: Confidence intervals
CPRD: Clinical Practice Research Datalink
DDD: Defined Daily Doses
GP: General practitioner
HF: Heart failure
HR: Hazard ratio
ICD: International Classification of Diseases
ICD-O: ICD for Oncology
MI: Myocardial infarction
NCDR: National Cancer Data Repository
NKCC: Na-K-2Cl (NKCC) transporter
ONS: Office of National Statistics
UK: United Kingdom
